# Rebamipide does not protect against naproxen-induced gastric damage: a randomized double-blind controlled trial

**DOI:** 10.1186/s12876-016-0472-x

**Published:** 2016-06-04

**Authors:** Thiago Gagliano-Jucá, Ronilson A. Moreno, Tiago Zaminelli, Mauro Napolitano, Antônio Frederico N. Magalhães, Aloísio Carvalhaes, Miriam S. Trevisan, John L. Wallace, Gilberto De Nucci

**Affiliations:** Institute of Biophysics Carlos Chagas Filho, Federal University of Rio de Janeiro (UFRJ), 21941-902 Rio de Janeiro, Brazil; Galeno Research Unit, Campinas, Brazil; Faculty of Medical Sciences, Metropolitan University of Santos, Santos, Brazil; Department of Pharmacology, Faculty of Medical Sciences, State University of Campinas (UNICAMP), Campinas, Brazil; Department of Pharmacology, ICB – University of Sao Paulo (USP), 05508-900 Sao Paulo, Brazil; Department of Gastroenterology, Faculty of Medical Sciences, State University of Campinas (UNICAMP), Campinas, Brazil; Brazilian Society for Digestive Endoscopy (SOBED), Sao Paulo, Brazil; Unicastelo Medical School, University Camilo Castelo Branco (UNICASTELO), Fernandopolis, Brazil; Department of Pharmacology, ICB – University of Sao Paulo (USP), Sao Paulo, Brazil

**Keywords:** NSAID, Histopathology, Human, ELISA, Endoscopy, Cryer score, Modified Lanza score

## Abstract

**Background:**

Rebamipide is a gastroprotective agent with promising results against gastric damage induced by non-steroidal anti-inflammatory drugs. The present study evaluated if rebamipide protects against naproxen-induced gastric damage in healthy volunteers. Changes in gastric PGE2 tissue concentration were also evaluated.

**Methods:**

After a preliminary endoscopy to rule out previous gastric macroscopic damage, twenty-four healthy volunteers of both sexes were divided into 2 groups. One group received sodium naproxen 550 mg b.i.d. plus placebo for 7 days, while the other group received sodium naproxen 550 mg b.i.d. plus rebamipide 100 mg b.i.d. At the end of treatment, a new endoscopy was performed. Gastric macroscopic damage was evaluated by the Cryer score and by the modified Lanza score. The primary outcome measure of the trial was the macroscopic damage observed in each treatment group at the end of treatment. Biopsies were collected at both endoscopies for PGE2 quantification and histopathological analysis (secondary outcomes). Tissue PGE2 was quantified by ELISA. The randomization sequence was generated using 3 blocks of 8 subjects each. Volunteers and endoscopists were blind to whether they were receiving rebamipide or placebo.

**Results:**

All recruited volunteers completed the trial. Sodium naproxen induced gastric damage in both groups. At the end of the study, median Cryer score was 4 in both groups (Difference = 0; 95%CI = −1 to 0; *p* = 0.728). In the placebo group, the mean tissue PGE2 concentration was 1005 ± 129 pg/mL before treatment and 241 ± 41 pg/mL after treatment (*p* < 0.001). In the rebamipide group, the mean tissue PGE2 concentration was 999 ± 109 pg/mL before treatment, and 168 ± 13 pg/mL after treatment (*p* < 0.001). There was no difference in mean tissue PGE2 between the two groups (difference = 5; 95%CI from −334.870 to 345.650; *p* = 0.975). No significant change was observed at the histopathological evaluation, despite the evident macroscopic damage induced by naproxen.

**Conclusion:**

Rebamipide does not protect against naproxen-induced gastric damage in healthy volunteers.

**Trial registration:**

ClinicalTrials.gov, NCT02632812. Registered 14 December 2015.

**Electronic supplementary material:**

The online version of this article (doi:10.1186/s12876-016-0472-x) contains supplementary material, which is available to authorized users.

## Background

Naproxen and other non-selective non-steroidal anti-inflammatory drugs (NSAIDs) induce gastrointestinal (GI) adverse events ranging from dyspepsia to upper and lower GI tract ulcers [1–3]. Naproxen 660 – 1000 mg per day administered for 2 to 14 days is used as a model for NSAID-induced gastroduodenal damage in healthy volunteers [4–8].

Rebamipide has been described as a GI mucosal protector agent with promising results in prophylaxis and treatment of GI ulcers caused by NSAIDs, *H. pylori* or endoscopic submucosal dissection [9–15]. Results from animal studies have suggested that the protective effects of rebamipide are attributable to stimulation of prostaglandin (PG) synthesis [16, 17]. In healthy volunteers, concomitant administration of rebamipide 100 mg with ibuprofen 600 mg t.i.d. for 7 consecutive days resulted in a mean gastric damage score of 1.3 ± 1.0, which was significantly lower than that of the control group (mean score of 2.9 ± 1.7), as assessed by the modified Lanza score (*p* = 0.032) [10]. In patients with rheumatic disease rebamipide 100 mg b.i.d. also showed a protective effect against NSAID-induced gastric damage [18].

The present study evaluated whether rebamipide could provide a protective effect against naproxen-induced gastric damage in healthy volunteers.

## Methods

### Volunteers

Volunteers of both sexes aged 18 years old or older without any significant cardiac, hepatic, renal, pulmonary, neurological, gastrointestinal or hematological diseases, as determined by their medical history, physical examination, and routine laboratory tests (hematology, blood biochemistry, urine analysis and fecal occult blood test), were invited to participate in this double-blind, randomized, parallel placebo-controlled phase II single-center trial. All subjects tested negative for hepatitis B and C (except for serologic scar), as well as HIV I and II, and were instructed to abstain from taking any medication including over-the-counter medication for 2 weeks prior to and during the study period. Pregnancy was an exclusion criterion. All women enrolled had negative β-HCG. All volunteers were informed about the aim and risks of the study by the clinical investigator and they all signed a written informed consent statement before entering the study. The study was performed according to the 2008 revised Declaration of Helsinki for bio-medical research involving human subjects and the 1996 rules of Good Clinical Practices. The study protocol was approved by the Committee of Research Ethics of the University of Sao Paulo, Sao Paulo, Brazil, on 26 November 2014 (before the initiation of the study procedures), and the study is registered at ClinicalTrials.gov, ID NCT02632812.

After performing a preliminary gastroduodenal endoscopy at the Vera Cruz Hospital (Campinas, Brazil) to rule-out prior macroscopic upper GI damage in the subjects, twenty-four healthy volunteers (12 men) were enrolled in the study. Other exclusion criteria were: achlorhydria (pH > 6.5); positive fecal occult blood test; drug abuse, including alcohol or tobacco.

### Treatment

Volunteers were randomly divided into 2 groups: group A received 550 mg of sodium naproxen (Flanax® - Bayer) plus an effervescent placebo (Biolab Indústria Farmacêutica Ltda.) diluted in 200 mL of water b.i.d. for 7 consecutive days; group B received 550 mg of sodium naproxen (Flanax® - Bayer) plus 100 mg of effervescent rebamipide (Biolab Indústria Farmacêutica Ltda.) diluted in 200 mL of water b.i.d. for 7 consecutive days. The randomization sequence was determined using the randomization generator available at www.randomization.com, using 3 blocks of 8 subjects each. Volunteers were blind to whether they were receiving rebamipide or placebo. To assure adherence, the drugs were administered by a member of the research team (also blind to treatment allocation) early in the morning and late in the evening for the whole duration of the study. A pharmacist was responsible for labeling treatments as A or B, and for breaking the code at the end of the study. Volunteers were required to be fasted in the morning and were only allowed to eat at least 2 h after administration of medications. In the evening, volunteers were required to be at least for 2 h without ingestion of food, and were also asked to avoid eating for the following 2 h after the dose.

Adverse reactions were individually evaluated. Causality relationship to the treatment was assessed with the aid of the Karch & Lasagna algorithm [19]. The necessity of interruption of treatment or specific therapy was individually assessed by the Principal Investigator depending on the severity of the reaction and the causality relationship to the treatment.

### Gastrointestinal damage evaluation

Gastroduodenal endoscopy was performed before the start of the study and in the morning of the 8th day of treatment in all volunteers. Exams were performed by a single endoscopist. The primary outcome measure of the trial was the macroscopic damage observed in each treatment group at the end of treatment. The number of mucosal erosions after treatment was counted and macroscopic mucosal injury scored according to Cryer [20] and to the modified Lanza score (MLS; Table 1) [9, 21]. Gastroduodenal ulcers were classified according to the Sakita-Miwa classification. Additionally, a second endoscopist retrospectively evaluated the exams, blind to the scores given by the first endoscopist. If there was any difference between the score given by the two endoscopists, the final score would be the mean value of the two numbers. Both endoscopists were blind to treatment group allocation of volunteers. In each exam 3 biopsies were collected from the gastric antrum and 3 from the gastric corpus for PGE2 quantification. Samples were immediately washed in phosphate buffer saline at pH 7.4 and stored individually in a dry Eppendorf tube and kept at −20 °C until analysis. Another 2 biopsies from each stomach region (4 total) were collected at each endoscopic procedure for histological analysis. Volunteers were questioned about GI symptoms at every dose. Changes in gastric PGE2 concentration, histopathological characteristics and GI symptoms at the end of the study compared to baseline were evaluated as secondary outcome measures. A more detailed questionnaire about GI symptoms such as heartburn, abdominal pain, fullness, nausea, vomiting, blood in stools or others was completed before receiving the first dose and at the last day of treatment. In the questionnaires, when any symptom was reported, the volunteer was asked to describe the intensity and frequency of the symptom over the previous 7 days. Routine laboratory tests were repeated at the end of treatment, including fecal occult blood test.

**Table 1:** Cryer and modified Lanza scores according to gastroduodenal mucosal injury

Score	Cryer score	Modified Lanza score
0	Normal or erythema	No hemorrhage or erosion observed
1	Any amount of submucosal hemorrhage or edema without erosions.	One or two hemorrhages or erosions observed in one gastric area.
2	1 erosion ± submucosal hemorrhage or edema.	Three to five hemorrhages or erosions observed in one gastric area.
3	2–4 erosions ± submucosal hemorrhage or edema.	Hemorrhages or erosions observed in two gastric areas; six or more hemorrhages or erosions observed in one gastric area, with the total number not exceeding ten in the entire stomach.
4	5 or more erosions and/or a single ulcer ± submucosal hemorrhage or edema.	Hemorrhages or erosions observed in three or more gastric areas; eleven or more hemorrhages or erosions observed widely in the entire stomach.
5	Multiple ulcers ± submucosal hemorrhage or edema.	Ulcer.

### PGE2 quantification

Tissue PGE2 concentration was quantified by enzyme-linked immunosorbent assay (ELISA) using Cayman Chemical Monoclonal Prostaglandin E_2_ EIA Kit (item number 514010). Briefly, biopsies from the gastric antrum and corpus collected in both endoscopic procedures were weighted and homogenized individually for 20 s by a Polytron in 1 mL of Na_3_PO_4_ buffer solution (pH = 7.4). Aliquots of 200 μL of the homogenate were placed in a dry bath at 37 °C for 20 min and then centrifuged at 20,800 *g* for 30 s. Supernatant (150 μL) was collected and diluted 1:100 (v/v) to be used in the ELISA. Each aliquot was assayed in duplicate and final PGE2 concentrations were adjusted for initial sample mass. Data are displayed as mean ± SEM. The person responsible for the quantification of PGE2 was blind to treatment group allocation of volunteers.

### Histopathological evaluation

Biopsies from gastric antrum and corpus were immediately put in formaldehyde after collection. Samples were stained in hematoxylin and eosin for characterization and graded according to a score described in Table **2**. Giemsa stain was used to diagnose *H. pylori* infection. The pathologist was also blind to treatments.

**Table 2:** Histhopathologic grade score developed for microscopic injury evaluation

Score	Findings
0	Normal gastric mucosa or mild chronic inflammation
1	Chronic gastritis without activity
2	Chronic gastritis with activity on antrum
3	Chronic gastritis with activity on the body
4	Chronic gastritis with activity on antrum and on the body

### Statistical analysis

For evaluation of statistical difference of tissue PGE2 between treatments, a *t*-test was performed. A paired *t* test was used to compare tissue PGE2 before and after treatments within each group. Endoscopic and histopathological scores between treatment groups were compared using the Mann–Whitney *U*-test. Gastrointestinal symptoms after treatments were compared by Fisher’s exact test. A p value <0.05 was considered significant. The sample size planned for the study was 24 volunteers of both sexes. This number was chosen based on the gastroprotective effects of rebamipide previously reported [9]. The calculation considered the Mann–Whitney *U* test analysis of the reported data on gastroduodenal damage scoring by MLS. The effect size observed (difference between treatments) was 67 % (median score after treatment of 3 and 1, in the placebo and rebamipide groups, respectively). Given a power of 80 % and a 0.05 chance of type 1 error, the original sample size estimation was 10 volunteers per group. The final number of 12 volunteers per group was chosen considering a 20 % rate of drop-out. Other studies with rebamipide in healthy volunteers also used similar sample sizes [10, 15].

## Results

All 24 volunteers enrolled completed all procedures of the study. The median age of men was 24 years (range 18 – 49 years), mean weight was 75.7 kg (61.0 – 97.0 kg), mean height was 175 cm (152 – 186 cm), and mean body mass index was 24.8 kg/m^2^ (20.2 – 28.7 kg/m^2^). Women had a median age of 24.5 years (range 20 – 42 years), mean weight was 69.5 kg (54.0 – 83.0 kg), mean height was 164 cm (153 – 170 cm), and mean body mass index was 25.9 kg/m^2^ (20.7 – 28.7 kg/m^2^). Three volunteers (two men) were not allowed to enroll in the study because of gastric damage observation in the preliminary endoscopy.

**Fig. 1:**
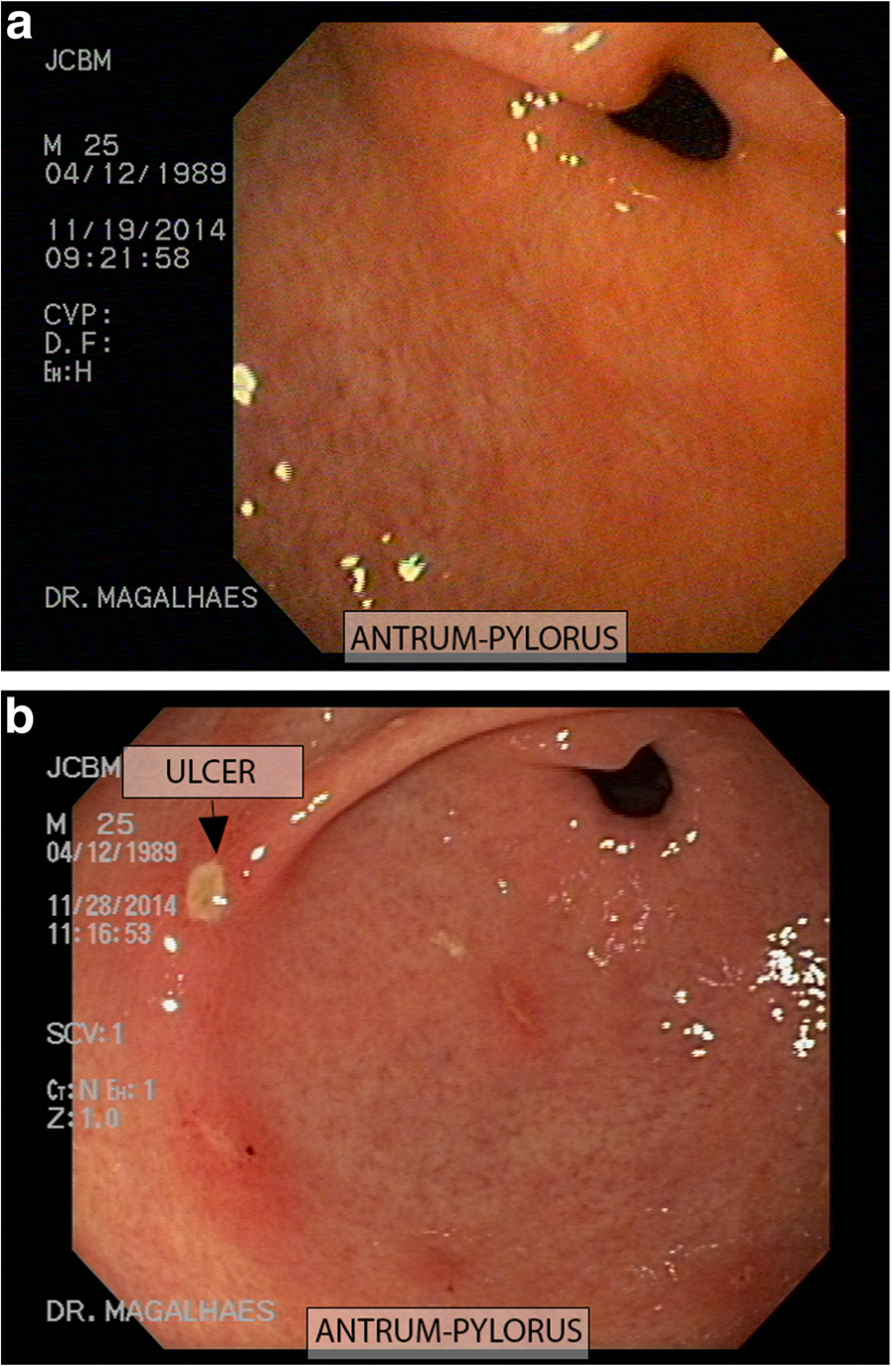
Photographic documentation of the endoscopic procedure in a volunteer in the rebamipide group (**a**) before and (**b**) after treatment

There was agreement between the endoscopists on the scores of all exams. Endoscopic findings and scores for each volunteer using the macroscopic scoring systems are summarized in Table 3. The median Cryer score was 4 in both placebo and rebamipide groups (Difference = 0; 95%CI = −1 to 0; *p* = 0.728). The median MLS was 4 in the placebo group and 3.5 in the rebamipide group (Difference = 0.5; 95%CI = −2 to 1; *p* = 0.822). In the placebo group 2 volunteers (16 %) presented GI ulcers (1 ulcer each), while 4 volunteers (32 %) in the rebamipide group had ulcers (*p* = 0.320). Figure 1a and b illustrate the macroscopic aspect of the gastric mucosa of volunteer 24 in the rebamipide group before treatment and at the end of the study.

**Table 3:** Summary of findings in the endoscopic procedure at the end of treatments

Volunteer #	Treatment	Scores before treatment	Cryer Score	Lanza Score	Antrum	Duodenum	Other
2	Placebo	0	3	2	4 erosions		
5	Placebo	0	2	1	1 erosion + submucousal hemorrage		
7	Placebo	0	4	4	>20 erosions		
8	Placebo	0	4	5	2 erosions + 1 ulcer (Sakita A2)		
10	Placebo	0	4	4	>10 erosions		
12	Placebo	0	4	5	10 erosions + 1 ulcer (Sakita A2)		
14	Placebo	0	4	2	5 erosions		
16	Placebo	0	4	4	20 erosions		
17	Placebo	0	4	4	10 erosions	3 erosions	
18	Placebo	0	4	4	10 erosions		
19	Placebo	0	4	4	10 erosions		
23	Placebo	0	4	4	10 erosions		
1	Rebamipide	0	4	4	20–25 erosions		
3	Rebamipide	0	4	4	10 erosions		
4	Rebamipide	0	4	5	10 erosions	1 ulcer (Sakita A2)	
6	Rebamipide	0	3	1	2 erosions + erythema		
9	Rebamipide	0	3	2	3 erosions		
11	Rebamipide	0	3	3	2 erosions		Submucousal hemorrhage in gastric fundus
13	Rebamipide	0	5	5	Small erosions + 2 ulcers (Sakita A2)		
15	Rebamipide	0	4	2	5 erosions + erythema		
20	Rebamipide	0	3	2	4 erosions		
21	Rebamipide	0	4	5			Pylorus with edema and 1 ulcer (Sakita A2)
22	Rebamipide	0	3	2	3 erosions		
24	Rebamipide	0	5	5	10 erosions + 4 ulcers (Sakita A2)

The incidence of GI symptoms in each treatment group is displayed in Table 4. Individual data regarding GI symptoms is available in Additional file 1: Table S1. There were no statistical differences between treatment groups at the end of the study. All fecal occult blood tests were negative at the end of treatment.

**Table 4:** Incidence of gastrointestinal symptoms

Gastrointestinal symptom	Placebo (*n* = 12)		Rebamipide (*n* = 12)		P value	
	Before	After	Before	After	Before	After
Any	3	5	5	3	0.67	0.33
Abdominal pain	2	3	3	2	0.50	0.50
Heartburn	2	2	3	1	0.50	0.50
Nausea	1	2	1	1	0.76	0.50
Intestinal cramps	0	1	1	0	0.50	0.50
Fullness	1	0	0	0	0.50	1.00

Incidence of gastrointestinal symptoms in each treatment group before the beginning and at the end of the study. *P* value was calculated comparing incidence of symptoms between placebo and rebamipide groups before and after treatments by Fisher’s exact test

### PGE2 quantification

In the placebo group, the mean tissue PGE2 concentration was 1005 ± 129 pg/mL before treatment, and 241 ± 41 pg/mL after treatment (difference = 764; 95%CI from 477 to 1051), which corresponded to a mean inhibition of PGE2 synthesis of 76.0 % (*p* < 0.001). In the rebamipide group, the mean tissue PGE2 concentration was 999 ± 109 pg/mL before treatment, and 168 ± 13 pg/mL after treatment (difference = 831.5; 95%CI from 612 to 1051), a mean inhibition of 83.2 % (*p* < 0.001). There was no difference between the two groups in PGE2 tissue concentration before treatments (difference = 5; 95%CI from −334.870 to 345.650; *p* = 0.975). The individual PGE2 concentration found for each volunteer before and after treatment is available in Additional file 2: Table S2.

### Histopathological evaluation

At the initial endoscopic procedure, median histopathological scores were 1 in both groups (Difference = 0; 95%CI = −3 to 1; *p* = 0.582). At the end of the study, median histopathological scores were also 1 in both groups (Difference = 0; 95%CI = −1 to 1; *p* = 0.997). At the initial endoscopy, 5 volunteers in each group were *H. pylori* positive. At the end of treatments, 6 subjects in each group were *H. pylori* positive. The individual histopathological score for each patient as well as *H. pylori* status before and after treatment is available in Additional file 3: Table S3.

## Discussion

The present study did not find any evidence of a gastroprotective effect of rebamipide on naproxen-induced gastroduodenal damage as assessed by endoscopic macroscopic evaluation. This result differs from previous findings, in which rebamipide treatment resulted in less severe gastric damage induced by NSAIDs [9, 10, 15]. One possible explanation for the different results found in the present work is that naproxen was more aggressive than others models of NSAID-induced gastric damage. To evaluate this possibility, previously published data was further analyzed. Treatment with indomethacin at 75 mg/day without rebamipide for 7 consecutive days resulted in a median MLS of 3. When treatment was associated with rebamipide 100 mg t.i.d., median MLS was 1 [9]. Treatment with ibuprofen 1800 mg/day for 7 consecutive days without rebamipide resulted in a median MLS of 3. When volunteers received rebamipide concomitantly with ibuprofen, median MLS was 2 [10]. By performing the Mann–Whitney *U*-test to compare MLS scores from the placebo group in the present study to those found in the placebo groups of the previously mentioned studies, no difference in gastric lesion scores among these 3 models of NSAID-induced gastric damage was observed (indomethacin vs. naproxen, *p* = 0.475; ibuprofen vs. naproxen, *p* = 0.343; indomethacin vs. ibuprofen, *p* = 1.0). However, since the characteristics of volunteers enrolled in the aforementioned studies are very different from those of the present study and trials designs are not the same, final conclusions cannot be drawn from these statistical analyses.

Since all the aforementioned studies evaluated Japanese or Korean subjects [9, 10, 13, 15, 18], population characteristics may have influenced the results. Higher gastric pH is known to be a protective factor in NSAID-induced gastric damage. The incidence of hypochlorhydria in the Japanese population is higher than in western countries, affecting more than 40 % of individuals over 50 year olds in Japan [22], while in the Unites States the incidence varies from 8 % in young adults to 11 % in the elderly [23, 24]. The higher gastric pH might facilitate the observation of the protective effect of rebamipide reported in Asian individuals. Another possibility for differences in rebamipide effects in NSAID-induced gastric damage is the genetic and dietary characteristics of the populations evaluated in each study.

One difference of the present study is that the daily dose of rebamipide was 200 mg, while most previous reports treated volunteers with 300 mg of rebamipide per day [9–15, 25, 26]. However, rebamipide at 200 mg/day in patients with rheumatic disease showed a protective effect in NSAID-induced gastroduodenal mucosal injury [18]. It is unlikely that a reduction in 33 % of the dose would abolish the supposed effect of rebamipide. A lower dosage of rebamipide was chosen due to safety concerns. Most of the safety data about rebamipide comes from studies in Asian populations. This is the first time a study evaluated effects of rebamipide in a Brazilian population.

Increased PG synthesis was previously suggested as a possible mechanism for mucosal protection of rebamipide [16, 17]. If so, rebamipide should not have a protective effect in NSAID-induced gastric damage through modulation of PG synthesis, since NSAIDs block the activity of cyclooxygenases.

In the present study, no significant histopathological change of the gastric mucosa was seen at the end of treatment in neither groups, despite the clear aggressiveness of naproxen observed on macroscopy. These results confirm previous reports that identified the histological features characteristic of NSAID users only in a subset of patients, and that microscopic findings do not correlate with macroscopical damage [27, 28]. This may reflect that the histological parameters used lack sufficient sensitivity or else, the mechanism responsible for the appearance of erosions and ulcers is independent of the degree of inflammation.

## Conclusion

Rebamipide 200 mg/day does not protect against naproxen-induced gastroduodenal damage in healthy volunteers.

## Abbreviations

ELISA, Enzyme-linked immunosorbent assay; GI, Gastrointestinal; MLS, Modifies Lanza score; NSAIDs, Non-steroidal anti-inflammatory drugs; PG, Prostaglandin.

## Additional files

Additional file 1: Table S1. Gastrointestinal symptoms occurrence in the previous 7 days as reported by each volunteer before the initiation and at the end of treatments. (DOCX 17 kb) Additional file 2: Table S2. Individual PGE2 concentration found at each assay for each gastric biopsy of each region evaluated, before and after treatment, for each volunteer. (DOCX 19 kb) Additional file 3: Table S3. Individual histopathological scores and *H. pylori* status before and after treatment for each volunteer. (DOCX 15 kb)
