# Supplementary material for: Rebamipide does not protect against naproxen-induced gastric damage: a randomized double-blind controlled trial
Source: BMC Gastroenterol. 2016 Jun 4;16:58. doi: 10.1186/s12876-016-0472-x (PMC4893238; doi:10.1186/s12876-016-0472-x)
Supplement: Additional file 1: Table S1. — Gastrointestinal symptoms occurrence in the previous 7 days as reported by each volunteer before the initiation and at the end of treatments. (DOCX 17 kb) [file 12876_2016_472_MOESM1_ESM.docx]

Additional file 1: Table S1 – Gastrointestinal symptoms occurrence in the previous 7 days as reported by each volunteer before the initiation and at the end of treatments.

|  | **Symptom** | **Volunteer Number** | | | | | | | | | | | | | | | | | | | | | | | |
| --- | --- | --- | --- | --- | --- | --- | --- | --- | --- | --- | --- | --- | --- | --- | --- | --- | --- | --- | --- | --- | --- | --- | --- | --- | --- |
|  |  | **1** | **2** | **3** | **4** | **5** | **6** | **7** | **8** | **9** | **10** | **11** | **12** | **13** | **14** | **15** | **16** | **17** | **18** | **19** | **20** | **21** | **22** | **23** | **24** |
| **Before** | Abdominal pain |  |  | X | X |  |  |  | X |  |  |  | X |  |  |  |  |  |  |  |  |  | X |  |  |
|  | Heartburn | X |  |  |  |  | X |  |  |  |  |  | X |  | X |  |  |  |  |  |  |  | X |  |  |
|  | Nausea |  |  |  |  |  |  |  |  |  |  |  |  |  | X |  |  |  |  |  |  |  | X |  |  |
|  | Intestinal cramps |  |  |  |  |  |  |  |  |  |  |  |  |  |  |  |  |  |  |  |  |  | X |  |  |
|  | Fullness |  |  |  |  |  |  |  |  |  |  |  |  |  | X |  |  |  |  |  |  |  |  |  |  |
| **After** | Abdominal pain |  | X | X |  |  |  |  | X |  | X |  |  |  |  |  |  |  |  |  |  |  |  |  | X |
|  | Heartburn |  | X |  |  |  | X |  |  |  |  |  | X |  |  |  |  |  |  |  |  |  |  |  |  |
|  | Nausea |  | X |  |  |  |  |  |  |  |  |  |  |  |  |  |  |  |  |  |  |  |  | X | X |
|  | Intestinal cramps |  |  |  |  |  |  |  |  |  | X |  |  |  |  |  |  |  |  |  |  |  |  |  |  |
|  | Fullness |  |  |  |  |  |  |  |  |  |  |  |  |  |  |  |  |  |  |  |  |  |  |  |  |

Volunteers 1, 3, 4, 6, 9, 11, 13, 15, 20, 21, 22 and 24 received 550 mg of sodium naproxen plus 100 mg of rebamipide twice a day for 7 consecutive days, while volunteers 2, 5, 7, 8, 10, 12, 14, 16, 17, 18, 19 and 23 received 550 mg of sodium naproxen plus placebo twice a day for 7 consecutive days.
