# Supplementary material for: Rebamipide does not protect against naproxen-induced gastric damage: a randomized double-blind controlled trial
Source: BMC Gastroenterol. 2016 Jun 4;16:58. doi: 10.1186/s12876-016-0472-x (PMC4893238; doi:10.1186/s12876-016-0472-x)
Supplement: Additional file 2: Table S2. — Individual PGE2 concentration found at each assay for each gastric biopsy of each region evaluated, before and after treatment, for each volunteer. (DOCX 19 kb) [file 12876_2016_472_MOESM2_ESM.docx]

Additional file 2: Table S2 – Individual PGE2 concentration found at each assay for each gastric biopsy of each region evaluated, before and after treatment, for each volunteer.

| **Volunteer number** | **Antrum - Before** | **Antrum - After** | **Corpus - Before** | **Corpus – After** |
| --- | --- | --- | --- | --- |
| 1 | 707.080 | 62.037 | 2850.646 | 340.500 |
| 1 | 1392.561 | 84.307 | 2067.442 | 188.583 |
| 1 | 1480.699 | 77.730 | 2232.419 | 181.588 |
| 2 | 891.722 | 61.706 | 830.697 | 189.621 |
| 2 | 820.075 | 54.062 | 792.398 | 118.157 |
| 2 | 1585.759 | 61.614 | 868.829 | 98.140 |
| 3 | 552.945 | 165.348 | 723.128 | 195.859 |
| 3 | 931.406 | 220.618 | 985.736 | 314.554 |
| 3 | 1123.416 | 125.514 | 585.243 | 158.860 |
| 4 | 831.188 | 211.678 | 754.644 | 287.316 |
| 4 | 567.020 | 185.618 | 1156.782 | 204.600 |
| 4 | 1136.637 | 214.158 | 1025.369 | 182.288 |
| 5 | 262.949 | 207.087 | 320.988 | 316.750 |
| 5 | 575.016 | 325.180 | 259.524 | 333.349 |
| 5 | 191.306 | 273.713 | 695.338 | 296.257 |
| 6 | 519.223 | 161.308 | 702.528 | 205.316 |
| 6 | 467.477 | 117.550 | 509.111 | 236.279 |
| 6 | 335.672 | 111.243 | 754.004 | 169.300 |
| 7 | 481.704 | 209.108 | 312.801 | 990.096 |
| 7 | 207.605 | 198.217 | 407.358 | 861.963 |
| 7 | 363.805 | 223.815 | 893.053 | 1118.449 |
| 8 | 1053.550 | 220.313 | 887.560 | 559.019 |
| 8 | 1080.349 | 211.382 | 1002.728 | 455.129 |
| 8 | 1405.187 | 164.175 | 1008.030 | 546.797 |
| 9 | 1370.398 | 225.762 | 1127.655 | 274.247 |
| 9 | 2008.761 | 152.144 | 653.584 | 275.912 |
| 9 | 1566.300 | 164.289 | 719.708 | 261.078 |
| 10 | 308.787 | 209.698 | 1118.808 | 398.277 |
| 10 | 226.190 | 318.795 | 922.260 | 641.265 |
| 10 | 367.016 | 69.373 | 1019.653 | 346.007 |
| 11 | 1524.442 | 205.585 | 2033.465 | 298.042 |
| 11 | 761.480 | 134.697 | 2754.343 | 211.077 |
| 11 | 877.024 | 139.881 | 777.400 | 272.713 |
| 12 | 1008.306 | 133.963 | 1691.035 | 219.089 |
| 12 | 1709.631 | 85.177 | 997.893 | 544.307 |
| 12 | 664.149 | 111.061 | 1236.820 | 238.078 |
| 13 | 759.713 | 195.477 | 1032.280 | 162.735 |
| 13 | 470.870 | 149.910 | 400.017 | 222.774 |
| 13 | 820.826 | 56.504 | 369.267 | 183.608 |
| 14 | 186.916 | 55.315 | 513.564 | 178.008 |
| 14 | 217.525 | 127.924 | 372.929 | 150.461 |
| 14 | 177.675 | 67.709 | 975.362 | 240.069 |
| 15 | 918.361 | 173.248 | 817.625 | 230.187 |
| 15 | 1423.563 | 310.619 | 1538.413 | 253.135 |
| 15 | 1251.284 | 48.890 | 539.615 | 155.973 |
| 16 | 2026.245 | 59.169 | 4081.267 | 190.063 |
| 16 | 672.996 | 124.048 | 1503.095 | 280.826 |
| 16 | 1117.783 | 1060.554 | 1465.184 | 257.332 |
| 17 | 208.671 | 114.888 | 1271.726 | 253.234 |
| 17 | 185.607 | 126.928 | 1794.850 | 250.840 |
| 17 | 320.567 | 93.520 | 954.771 | 101.681 |
| 18 | 911.780 | 74.274 | 846.158 | 149.815 |
| 18 | 662.843 | 73.249 | 957.398 | 123.136 |
| 18 | 2284.297 | 80.637 | 2186.647 | 235.200 |
| 19 | 38.293 | 96.295 | 2772.583 | 71.971 |
| 19 | 1226.709 | 94.836 | 2375.770 | 112.064 |
| 19 | 1314.839 | 49.972 | 2723.506 | 401.663 |
| 20 | 1606.537 | 100.225 | 1871.370 | 206.972 |
| 20 | 1896.501 | 78.884 | 1595.973 | 147.436 |
| 20 | 1523.415 | 113.723 | 2274.115 | 103.732 |
| 21 | 961.850 | 92.885 | 507.110 | 194.488 |
| 21 | 550.857 | 78.378 | 687.679 | 133.869 |
| 21 | 435.062 | 134.252 | 280.885 | 165.863 |
| 22 | 264.850 | 53.872 | 317.728 | 63.422 |
| 22 | 344.976 | 44.506 | 1485.621 | 55.720 |
| 22 | 654.343 | 81.441 | 867.762 | 116.137 |
| 23 | 308.316 | 92.004 | 547.714 | 164.729 |
| 23 | 708.940 | 113.373 | 2178.797 | 98.653 |
| 23 | 886.451 | 48.440 | 2898.958 | 131.088 |
| 24 | 395.110 | 57.943 | 856.675 | 323.540 |
| 24 | 293.998 | 66.834 | 612.895 | 211.562 |
| 24 | 238.601 | 48.573 | 524.823 | 256.746 |

Volunteers 1, 3, 4, 6, 9, 11, 13, 15, 20, 21, 22 and 24 received 550 mg of sodium naproxen plus 100 mg of rebamipide twice a day for 7 consecutive days, while volunteers 2, 5, 7, 8, 10, 12, 14, 16, 17, 18, 19 and 23 received 550 mg of sodium naproxen plus placebo twice a day for 7 consecutive days.
