# Supplementary material for: Rebamipide does not protect against naproxen-induced gastric damage: a randomized double-blind controlled trial
Source: BMC Gastroenterol. 2016 Jun 4;16:58. doi: 10.1186/s12876-016-0472-x (PMC4893238; doi:10.1186/s12876-016-0472-x)
Supplement: Additional file 3: Table S3. — Individual histopathological scores and H. pylori status before and after treatment for each volunteer. (DOCX 15 kb) [file 12876_2016_472_MOESM3_ESM.docx]

| **Volunteer number** | **Histopathol Before** | ***H. pylori* Before** | **Histopathol After** | ***H. pylori* After** |
| --- | --- | --- | --- | --- |
| **1** | 4 | + | 4 | + |
| **2** | 4 | + | 4 | + |
| **3** | 0 | - | 0 | - |
| **4** | 0 | - | 0 | - |
| **5** | 1 | - | 1 | - |
| **6** | 1 | - | 1 | - |
| **7** | 0 | - | 0 | - |
| **8** | 0 | - | 0 | - |
| **9** | 1 | + | 2 | + |
| **10** | 1 | - | 1 | - |
| **11** | 1 | - | 4 | + |
| **12** | 4 | + | 2 | + |
| **13** | 0 | - | 0 | - |
| **14** | 0 | - | 0 | - |
| **15** | 0 | - | 0 | - |
| **16** | 4 | + | 4 | + |
| **17** | 0 | - | 1 | - |
| **18** | 4 | + | 4 | + |
| **19** | 1 | - | 1 | + |
| **20** | 1 | + | 4 | + |
| **21** | 2 | + | 1 | + |
| **22** | 1 | + | 1 | + |
| **23** | 1 | + | 1 | + |
| **24** | 1 | - | 2 | - |

Additional file 3: Table S3 – Individual histopathological scores and *H. pylori* status before and after treatment for each volunteer.

Volunteers 1, 3, 4, 6, 9, 11, 13, 15, 20, 21, 22 and 24 received 550 mg of sodium naproxen plus 100 mg of rebamipide twice a day for 7 consecutive days, while volunteers 2, 5, 7, 8, 10, 12, 14, 16, 17, 18, 19 and 23 received 550 mg of sodium naproxen plus placebo twice a day for 7 consecutive days.
